# Whole Genome Sequencing of *Enterovirus species C* Isolates by High-Throughput Sequencing: Development of Generic Primers

**DOI:** 10.3389/fmicb.2016.01294

**Published:** 2016-08-26

**Authors:** Maël Bessaud, Serge A. Sadeuh-Mba, Marie-Line Joffret, Richter Razafindratsimandresy, Patsy Polston, Romain Volle, Mala Rakoto-Andrianarivelo, Bruno Blondel, Richard Njouom, Francis Delpeyroux

**Affiliations:** ^1^Unité de Biologie des Virus Entériques, Institut PasteurParis, France; ^2^Institut National de la Santé et de la Recherche Médicale, U994Paris, France; ^3^WHO Collaborating Center for Research on Enteroviruses and Viral Vaccines, Institut PasteurParis, France; ^4^Centre Pasteur du Cameroun, Service de VirologieYaoundé, Cameroon; ^5^Unité de virologie, Institut Pasteur de MadagascarAntananarivo, Madagascar; ^6^Centre d'infectiologie Charles-Mérieux, Université AnkatsoAntananarivo, Madagascar

**Keywords:** *Enterovirus species C*, poliovirus, high-throughput sequencing, recombination, *de novo* assembly

## Abstract

Enteroviruses are among the most common viruses infecting humans and can cause diverse clinical syndromes ranging from minor febrile illness to severe and potentially fatal diseases. *Enterovirus species C* (EV-C) consists of more than 20 types, among which the three serotypes of polioviruses, the etiological agents of poliomyelitis, are included. Biodiversity and evolution of EV-C genomes are shaped by frequent recombination events. Therefore, identification and characterization of circulating EV-C strains require the sequencing of different genomic regions. A simple method was developed to quickly sequence the entire genome of EV-C isolates. Four overlapping fragments were produced separately by RT-PCR performed with generic primers. The four amplicons were then pooled and purified prior to being sequenced by a high-throughput technique. The method was assessed on a panel of EV-Cs belonging to a wide-range of types. It can be used to determine full-length genome sequences through *de novo* assembly of thousands of reads. It was also able to discriminate reads from closely related viruses in mixtures. By decreasing the workload compared to classical Sanger-based techniques, this method will serve as a precious tool for sequencing large panels of EV-Cs isolated in cell cultures during environmental surveillance or from patients, including vaccine-derived polioviruses.

## Introduction

The members of the *Enterovirus species C* (EV-C), genus *Enterovirus*, family *Picornaviridae*, are non-enveloped viruses with a single positive strand RNA genome. The virions contain one copy of the genome, which is about 7500 nucleotides in length and consists of two untranslated regions (5′- and 3′-UTR) flanking a unique large open reading frame. The polyprotein encoded by this open reading frame is first cleaved into three precursors (P1–P3) that are subsequently cleaved into functional proteins. P1 gives rise to the four structural capsid proteins (VP1–VP4) while P2 and P3 generate non-structural proteins involved in the viral cycle. Enteroviruses cause a wide spectrum of human diseases, with clinical signs ranging from mild febrile illness, such as the common cold, to severe forms, such as acute haemorrhagic conjunctivitis, myocarditis, encephalitis, and acute flaccid paralysis (Tapparel et al., 2013).

Currently, more than 20 types of EV-Cs have been identified (Knowles et al., 2012), including the three serotypes of poliovirus (PV-1 to −3) that can induce severe and potentially fatal cases of poliomyelitis in humans. Typing of enteroviruses relies on molecular characterization of the capsid-encoding region (Blomqvist and Roivainen, 2016). For this purpose, many generic assays were developed to amplify genomic fragments within the VP4-, VP2-, or VP1-encoding regions by RT-PCR and to sequence the resulting amplicons. The sequences of clinical or field viruses can be compared to those of prototype strains in order to determine their respective types.

Besides typing, molecular characterization of circulating EV-Cs generally implies sequencing of untranslated or non-structural genomic regions, or even full-length sequencing of the genome. Indeed, recombination events between enteroviruses are known to be very frequent, thus leading to complex ecosystems of co-circulating viruses featuring mosaic genomes (Savolainen-Kopra and Blomqvist, 2010; Combelas et al., 2011; Kyriakopoulou et al., 2015). As recombination constitutes a powerful force that drives enterovirus evolution, sequencing of different genomic regions of isolates is crucial to detect recombination events. Such events are revealed by incongruent clustering of genetic sequences in phylogenetic trees, depending on the studied genomic regions.

Generic assays have been developed to amplify non-structural regions of EV genomes, particularly the 5′-UTR and the 2C- and the 3D-encoding regions (Bessaud et al., 2008). These wide-range assays can be used to characterize portions of the genome of EV-Cs but determining the full-length genomic sequences requires additional sequencing steps to bridge the gaps between the sequences determined through generic assays. These steps require the usage of specific primers that have to be designed for each isolate and can be very labor-intensive when numerous isolates of many types are studied.

In order to facilitate the full-length sequencing of EV-C isolates, a simple method was developed, which allowed the amplification of the whole genome by RT-PCR using four generic primer pairs. Four overlapping fragments were produced by performing four separate PCR reactions on cDNAs generated through a single RT reaction. The four amplicons were then pooled and purified prior to sequencing by high-throughput sequencing methods.

The amplification step was assessed on a panel of prototype and field strains whose genome had been previously sequenced by the Sanger method. Sequencing data was analyzed by mapping the reads against reference sequences and by *de novo* assembly. This method was able to determine the full-length genomic sequences of EV-C isolates with high sensitivity and to discriminate the different genomic sequences within mixtures of viruses.

## Materials and methods

### Viruses

Twenty-two viruses were used for this study (Table 1). The prototype viruses are available on the Pasteur Institute collection (*Centre de ressources biologiques de l'Institut Pasteur*, Paris, France). Field isolates originated from stool samples collected in Madagascar in 2002 (Rakoto-Andrianarivelo et al., 2005, 2007) and in Chad in 2008–2009 (Sadeuh-Mba et al., 2013). The whole genomic sequence of these isolates were previously determined by the Sanger method (Bessaud et al., 2011).

**Table 1:** **Viruses used in the study**.

**Type**	**Strain or isolate**	**Origin**	**Cycle threshold^a^**
CV-A1	Tompkins	Prototype strain	14.5 ± 0.2
CV-A11	Belgium	Prototype strain	16.6 ± 0.2
CV-A11	G9	Prototype strain	14.9 ± 0.7
CV-A11	66122	From Rakoto-Andrianarivelo et al. (2007)	15.6 ± 0.1
CV-A11	66990	From Rakoto-Andrianarivelo et al. (2007)	15.9 ± 0.1
CV-A13	Flores	Prototype strain	15.7 ± 0.3
CV-A13	G13	Prototype strain	23.7 ± 0.1
CV-A13	67900	From Rakoto-Andrianarivelo et al. (2007)	17.1 ± 0.4
CV-A13	67001	From Rakoto-Andrianarivelo et al. (2007)	13.3 ± 0.2
CV-A17	G12	Prototype strain	13.8 ± 0.3
CV-A17	67591	From Rakoto-Andrianarivelo et al. (2007)	14.4 ± 0.9
CV-A17	68154	From Rakoto-Andrianarivelo et al. (2007)	15.2 ± 0.2
CV-A19	NIH-8663	Prototype strain	23.2 ± 1.0
CV-A20	Cecil	Prototype strain	16.0 ± 0.1
CV-A20	IH35	Prototype strain	13.5 ± 0.4
CV-A20	Tulane	Prototype strain	16.1 ± 0.1
CV-A21	Coe	Prototype strain	17.7 ± 0.3
EV-C95	T08-083	From Sadeuh-Mba et al. (2013)	14.5 ± 0.5
EV-C99	68229	From Rakoto-Andrianarivelo et al. (2007)	11.5 ± 0.1
PV-1	Sabin	Vaccine strain	15.6 ± 0.1
PV-2	Sabin	Vaccine strain	17.4 ± 0.3
PV-3	Sabin	Vaccine strain	20.9 ± 0.1

a *Cycle threshold values obtained by a pan-enterovirus real-time RT-PCR assay performed on the extracted RNAs*.

All work with infectious viruses was carried out in a BSL-2 facility. All viruses were grown in HEp-2c cell monolayers in DMEM supplemented with 2% fetal calf serum and 2 mM L-glutamine at 37°C, except coxsackievirus (CV) A1 Tompkins and CV-A19 NIH-8663, see below. These latter cannot be propagated in cell lines but can infect suckling mice. RNA of these two viruses was extracted from brains of mice inoculated intracerebrally. These brains were retrieved from the laboratory collection and no mice were used during this study. Brains were crushed in PBS before RNA extraction.

### Sequence analysis and primer design

EV-C full-length nucleotide sequences retrieved from the GenBank database were aligned using CLC Main Workbench 7.6.4 software (CLC bio). Eight degenerated primers were designed to target conserved genomic regions (Table 2).

**Table 2:** **Primers used in this study**.

**Step**	**Name**	**5′ → 3′ sequence**	**Genome position^a^**
cDNA synthesis	heptaN	NNNNNNN	
PCR	C004	TTAAAACAGCYYKDGGGTTG	1–20
	C005	CCGAATYAAARRAAAATTTACCC	7437–7415
	C008	CARTTYAAGASCAARCAYCG	4459–4478
	C009	ACCATYTGRCARAANARYTTCA	4703–4682
	C018	ATGYTNGGNACNCAYNTNATHTGGGA	2212–2237
	C019	CCYTGYTCCATNRCYTCHTCYTC	3833–3811
	C021	TCDGGNARYTTCCACCACCA	1205–1186
	C022	GARGCNTGYGGDTAYAGYGA	967–986

a *Relative to PV-2 strain Sabin*.

### RNA extraction

Viral RNA was extracted from 250 μL of culture supernatants or clarified brain extracts using the High Pure Viral RNA kit (Roche Diagnostics, Meylan, France), following the manufacturer's instructions.

In order to check the RNA extraction step, a one-step real-time RT-PCR assay was performed on extraction products using a pan-enterovirus generic assay previously described (Monpoeho et al., 2000).

### Synthesis of the four overlapping amplicons by 2-step RT-PCR

**Figure 1:**
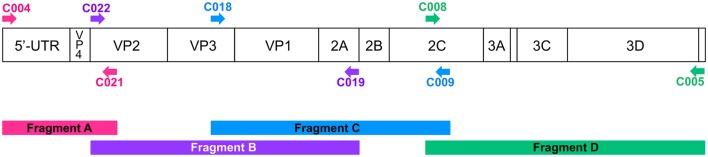
**Schematic representation of the locations of the primers used for the PCR step**. The upper scheme represents the organization of the enterovirus genomic RNA. Arrows indicate the sites targeted by the primers. The RT-PCR products are colored according to the corresponding primer pair.

For each virus, four overlapping DNA fragments were produced by RT-PCR (Figure 1). cDNA synthesis was performed as previously described (Bessaud et al., 2008). The reaction mixture contained 5 μL of purified viral RNA, 2 μL of 5X First-Strand Buffer, 0.01 M dithiothreitol (1 μL), 100 ng of the random primers heptaN (1 μL), 10 nmol of each dNTP (1 μL of a 10 mM mixture), and 100 U of SuperScript II (0.5 μL). The RT reaction mixture was incubated at 25°C for 10 min, 42°C for 45 min and 95°C for 5 min.

The cDNA was then used as a template for amplification in four PCRs carried out in a final volume of 50 μL that included 5 μL of 10X PCR Buffer w/o MgCl_2_, 1.5 mM of MgCl_2_, 10 nmol of each dNTP, 50 pmol of each primer, 2 μL of cDNA, and 2.5 U of Platinum *Taq* DNA polymerase (Invitrogen). The thermocycler profile was 2 min at 94°C followed by 30 cycles of 30 s at 94°C, 30 s at 55°C, and 3 min at 72°C.

Ten microliters of each PCR product were analyzed on ethidium bromide-stained agarose gels. For each virus, the four PCR products were pooled, purified on silica columns (Wizard SV Gel and PCR Clean-Up System, Promega) and eluted in 30 μL of water. No purification of the amplicons was performed by gel-excision, even when additional bands were detected on ethidium bromide-stained agarose gels.

### Assay sensitivity

The sensitivity of the assay was evaluated using serial three-fold dilutions of a cell culture supernatant infected by CV-A13 isolate 67001 whose titer was determined according to the WHO standard protocol (Anonymous, 2004). To maintain the same amount of cellular nucleic acids across dilutions, dilutions were prepared using a supernatant of confluent non-infected HEp-2c cell monolayer that was frozen and thawed twice and then clarified by centrifugation. RNA was extracted from 250 μL of each dilution and subjected to real-time RT-PCR as described in Section RNA Extraction, and amplified by RT and PCR as described in Section Synthesis of the Four Overlapping Amplicons by 2-Step RT-PCR.

### Detection of virus mixtures

Four mixtures were prepared using cell culture supernatants of CV-A13 isolates 67900 and 67001 whose titer was determined according to the WHO standard protocol (Anonymous, 2004). Titers were adjusted to 10^7.5^ TCID_50_.mL^−1^ before mixing. Different mixtures were prepared with 67900/67001 titer ratios ranging from 1:1 to 10:1. RNA was extracted from 250 μL of these mixtures and subjected to RT and PCR as described in Section Synthesis of the Four Overlapping Amplicons by 2-Step RT-PCR.

### Sequencing process

DNA concentration of the purified RT-PCR products was determined by using a VarioskanLux (ThermoScientific). Libraries were built using 1 ng of DNA with the Nextera XT DNA Library Preparation kit in a SureCycler 8800 thermocycler (Agilent). After purification on AMPure beads (Beckman), the libraries were controlled using the High Sensitivity D1000 assay (Agilent) on a TapeStation 2200. Sizing was achieved by electrophoresis on a PippinPrep System with the PippinPrep kit CDF1510 (Ozyme). Finally, the libraries were quantified using the KAPA Quantification kit on a LightCycler 96 System (Roche). Sequencing was performed on a NextSeq500 with the HighOutput or MidOutput kits. All kits were used following manufacturer's instructions.

### Data analysis

#### Trimming

Reads were demultiplexed followed by the removal of tags and adaptors. After importation in CLC Genomics Workbench 8.5 (CLCbio), reads were trimmed using the following parameters: Trim quality score limit = 0.01; Trim ambiguous nucleotides with Maximum number of ambiguities = 1. To avoid the presence of primer sequences in the reads, the 26 5′- and 3′-terminal nucleotides were removed. After trimming, reads shorter than 50 nucleotides were discarded.

#### Mapping of the reads to a reference

For each sample, the trimmed reads were mapped against the reference sequence of the corresponding virus. The sequences used as references were the sequences previously determined by the Sanger method. The entire genome of all field isolates were previously sequenced in our laboratory (Bessaud et al., 2011); for prototype strains, the sequences used as references were retrieved from the Genbank database. Mapping was achieved with CLC Genomics Workbench 8.5 by using the following parameters: Mismatch cost = 10; Insertion cost = 3; Deletion cost = 3; Length fraction = 0.5; Similarity fraction = 0.95.

#### *De novo* assembly

For each sample, the trimmed reads were assembled without any reference using CLC Genomics Workbench 8.5 with the following parameters: Mismatch cost = 2; Insertion cost = 2; Deletion cost = 2; Length fraction = 0.5; Similarity fraction = 0.95.

All contigs longer than 200 nucleotides were submitted to BLAST analysis (Mount, 2007). Virus contigs were compared with the reference sequence of the corresponding virus determined by the Sanger method through alignment.

## Results

### Sequence analysis and primer design

Eight degenerated primers were designed to target nucleotide sequences that were conserved among EV-Cs (Figure 1). C004 and C005 target the extremity of the 5′ and 3′ non-coding region, respectively. C018, C019, and C008 match with conserved regions already targeted by pan-EV molecular assays (Caro et al., 2001; Nix et al., 2006; Bessaud et al., 2008) within the VP3 gene, at the 2A/2B junction, and within the 2C *cis*-acting replication element (Cordey et al., 2008), respectively. The three other primers, C021, C022, and C009 match with VP2 and 2C sequences.

The primer pairs C004/C021, C022/C019, C018/C009, and C008/C005 were used to produce four overlapping DNA fragments (fragments A–D) that span the entire viral genome. Fragments ranged from ~1200 to ~3000 nucleotides in length. Overlapping regions were approximately 240-nt-long for fragments A and B and fragments C and D (Figure 1). The overlapping region for fragments B and C was longer than 1600 nt.

### RT-PCR amplification

The four primer pairs C004/C021, C022/C019, C018/C009, and C008/C005 were tested on prototype and field strains representative of 12 EV-C types (Table 1). This panel included the three PV serotypes and non-polio types commonly isolated during environmental or epidemiological surveillance, such as CV-A11, CV-A13, CV-A17, CV-A20, CV-A21, and EV-C99. One field strain belonged to the type EV-C95 that was recently discovered (Junttila et al., 2015) and of which few isolates were reported in Africa. The panel also included the prototype strains of CV-A1 and CV-A19, two types that are uncultivable on cell lines (Brown et al., 2003). Overall, 22 viruses were used to assess the efficiency of the primers. After RNA extraction, detection of viral RNA by real-time RT-PCR resulted in cycle threshold values ranging from 11.5 ± 0.1 to 23.7 ± 0.1 (Table 1).

Following the RT step with random primers performed on viral RNA extracted from infected cell culture supernatants (or from crushed brains of mice infected by CV-A1 or CV-A19), the PCR performed with the four primer pairs produced gel bands at the expected size for all the viruses (data not shown). Some additional bands were also observed for certain RT-PCR products. Among our panel, CV-A19 NIH-8663 RNA gave the weakest bands on ethidium bromide-stained gel after the RT-PCR step. It is not possible to exclude the hypothesis that the four primer pairs had a low efficiency in amplifying the genome of this virus through PCR because of mismatches. Nonetheless, since the RNA of this virus was extracted from a mouse brain collected more than 20 years ago, this result is more likely due to the low amount of full-length virus RNA in this sample.

### Results of sequencing

The products of RT-PCR amplification of non-polio EV were sequenced by Illumina technology. Due to French regulations relating to PV containment, the sequencing platform was not allowed to handle the PV RT-PCR products.

For each sample, the four RT-PCR fragments were produced separately and then pooled prior to purification. Sequencing resulted in a list of reads of which most were ~100 nucleotide-long after trimming. Because all of the samples were not sequenced during the same run, the number of reads varied from sample to sample. Thus, the number of reads after trimming ranged from 76,802 to 714,322 (Table 3).

**Table 3:** **Overview of the results of the bioinformatics analysis performed on sequencing data**.

**Strain or isolate**	**Total number of reads**	**Mapping on reference sequence**		***De novo* assembly**	
		**Number of mapped reads (%^a^)**	**Average depth**	**Number of reads in the virus contig (%^b^)**	**Number of host contigs**
**CV-A1**					
Tompkins	391,846	380,207 (97%)	7032	379,991 (96%)	200
**CV-A11**					
Belgium	373,748	337,637 (90%)	6214	337,708 (90%)	232
G9	121,080	119,260 (98%)	2310	120,181 (99%)	16
66122	76,802	76,342 (99%)	982	76,290 (99%)	3
66990	121,784	114,961 (94%)	2277	120,712 (99%)	10
**CV-A13**					
Flores	88,094	86,883 (97%)	1608	81,912 (93%)	6
G13	101,372	98,056 (96%)	1902	100,746 (99%)	12
67900	175,426	174,488 (99%)	3382	154,526 (88%)	1
67001	189,890	185,674 (97%)	3689	188,303 (99%)	0
**CV-A17**					
G12	554,763	553,436 (99%)	10,404	553,221 (99%)	28
67591	421,668	367,691 (87%)	4741	365,019 (87%)	197
68154	84,786	83,710 (98%)	1548	83,251 (98%)	14
**CV-A19**					
NIH-8663	374,976	153,262 (40%)	2958	152,980 (40%)	783
**CV-A20**					
Cecil	619,194	616,252 (99%)	11,525	615,858 (99%)	59
IH35	698,498	630,711 (99%)	11,657	632,064 (99%)	39
Tulane	714,322	712,310 (99%)	13,336	712,445 (99%)	21
**CV-A21**					
Coe	570,328	525,606 (92%)	9608	565,659 (99%)	115
**EV-C95**					
T08-083	96,460	96,059 (99%)	1776	96,110 (99%)	7
**EV-C99**					
68229	575,410	573,084 (99%)	10,435	572,958 (99%)	19

a *These percentages indicate the proportion of total reads that matched with the reference sequence*.

b *These percentages indicate the proportion of total reads that were included in the virus contig through de novo assembly*.

### Bioinformatics analysis

Raw data generated by high-throughput sequencing was analyzed by two different methods commonly used for virus characterization.

The first method consists in mapping the reads against a reference sequence. This method can be used to re-sequence isolates or to detect single-point mutations that appear when a given virus is propagated under different conditions (e.g., various host species, various cell lines or presence of antiviral molecules, for example).

The second method consists in constructing contigs through *de novo* assembly (i.e., without any reference sequence to drive the assembly). This method can be used to determine the full-length sequence of viruses isolated from clinical or environmental samples.

#### Mapping on reference sequence

For each sample, reads were mapped against the corresponding sequence previously determined by the Sanger method (Table 3). For all samples except one, more than 87% of the reads mapped against the reference sequence. The only exception was CV-A19 NIH-8663, a non-cultivable virus, in which only 40% of the reads mapped to the reference.

For all samples, the reference sequence was fully covered without any gaps (breadth of coverage >99.0%). The breadth of coverage never reached 100.0% because the sequences corresponding to the outer primers C004 and C005 and the few nucleotides located downstream C005 were absent in the final contig.

**Figure 2:**
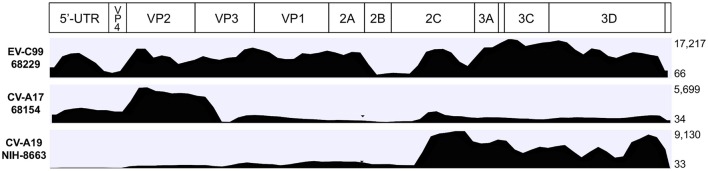
**Coverage depth observed for three samples after mapping on their respective reference sequence**. Coverage depth is the number of times a base is sequenced. On the right side of each graph, the minimum and maximum coverage depth values are indicated.

Average depth of coverage, which is the average number of times a base is sequenced, was very high (>980) but varied greatly from sample to sample (Table 3), depending on the total number of reads (correlation coefficient >0.999). Coverage depth was heterogeneous along the mapping (Figure 2) but was high enough to deduce an unambiguous consensus sequence even for samples with a low number of reads (CV-A11 66122 or CV-A17 68154 for instance) or a low proportion of mapping reads (CV-A19 NIH-8663).

The consensus sequences of field strains deduced from mapping were compared to the consensus sequences determined by the Sanger method using RNA extracted from the same cell culture supernatants. For each virus, the two consensus sequences were identical indicating that high-throughput sequencing did not introduced nucleotide changes in the consensus sequences compared to the Sanger method.

#### *De novo* assembly

In order to assess the ability of the method to determine full-length sequences of the original isolates, the trimmed reads of each sample were assembled without using a reference.

For all samples, the *de novo* assembly tool of CLC Genomics Workbench succeeded in assembling the virus genome into a unique contig that covered the entire genome between the outer primers C004 and C005. The number of reads included in the final full-length contigs was virtually similar to the number of reads previously found to map against the reference (Table 3). The consensus sequence deduced from *de novo* assembly was identical to the consensus sequence already determined by mapping.

For most samples, besides the viral contigs, *de novo* assembly produced additional contigs that were identified as human sequences (or mouse sequences for CV-A1 and CV-A19 samples) by BLAST analysis. These contigs were from non-specific amplification of cellular nucleic acids during the RT-PCR process or from contamination of the viral RNA with cellular DNA during extraction. The number of such contigs varied tremendously from sample to sample, ranging from 0 to 783 (Table 3). Nonetheless, contaminating nucleic acids did not compromise the proper assembly of the viral genome, even for the CV-A19 sample that contained mostly non-viral reads.

### Sensitivity of the assay

In order to evaluate the sensitivity of the assay, three-fold serial dilutions of a CV-A13 67001 supernatant at 10^7.8^ TCID_50_.mL^−1^ were prepared. After RNA extraction, detection of viral RNA by real-time RT-PCR showed positive results for extracts from the first 15 dilutions (Table 4).

**Table 4:** **Evaluation of the sensitivity of the assay**.

**Dilution factor**	**Viral titer (TCID_50_.mL^−1^)**	**RT-PCR cycle threshold**	**DNA concentration (ng.μL^−1^) ^b^**	**Number of reads**	**Number of reads mapping against the reference (% ^c^)**	**Construction of a full-length contig through *de novo* assembly**
3^0^	10^7.8^	16.2 ± 0.1	>200.0	197,190	196,993 (99.9%)	Yes
3^1^	10^7.3^	17.9 ± 0.4	90.4	143,770	143,125 (99.5%)	Yes
3^2^	10^6.8^	20.1 ± 0.4	76.7	140,554	138,103 (98.2%)	Yes
3^3^	10^6.4^	21.6 ± 0.2	18.7	112,070	104,730 (93.4%)	Yes
3^4^	10^5.9^	23.3 ±0.1	6.8	190,346	172,273 (90.5%)	Yes
3^5^	10^5.4^	25.1 ± 0.1	5.5	316,336	218,511 (69.1%)	Yes
3^6^	10^4.9^	26.7 ± 0.1	3.9	216,594	117,302 (54.1%)	Yes
3^7^	10^4.5^	28.4 ± 0.2	3.4	117,920	48,054 (27.0%)	Yes
3^8^	10^4.0^	30.0 ± 0.2	3.8	130,334	14,828 (11.3%)	Yes
3^9^	10^3.5^	31.6 ± 0.1	2.8	88,930	2282 (2.5%)	Yes
3^10^	10^3.0^	33.4 ± 0.2	2.5	119,950	1109 (0.9%)	No
3^11^	10^2.6^	35.2 ± 0.6	3.1	104,470	511 (0.4%)	No
3^12^	10^2.1^	36.1 ± 0.3	2.7	124,876	297 (0.2%)	No
3^13^	10^1.6^	37.5 ± 0.6	1.1	105,656	208 (0.2%)	No
3^14^	10^1.1^	> 38.0^a^	1.5	176,260	314 (0.2%)	No
3^15^	10^0.6^	> 39.0^a^	1.1	111,890	228 (0.2%)	No

a *Only one well of the triplicate assay showed a detectable increase in fluorescence*.

b *After pooling of the four RT-PCR products and purification, the DNA concentration was measured on a VarioskanLux spectrophotometer*.

c *These percentages indicate the proportion of total reads that matched with the reference sequence*.

**Figure 3:**
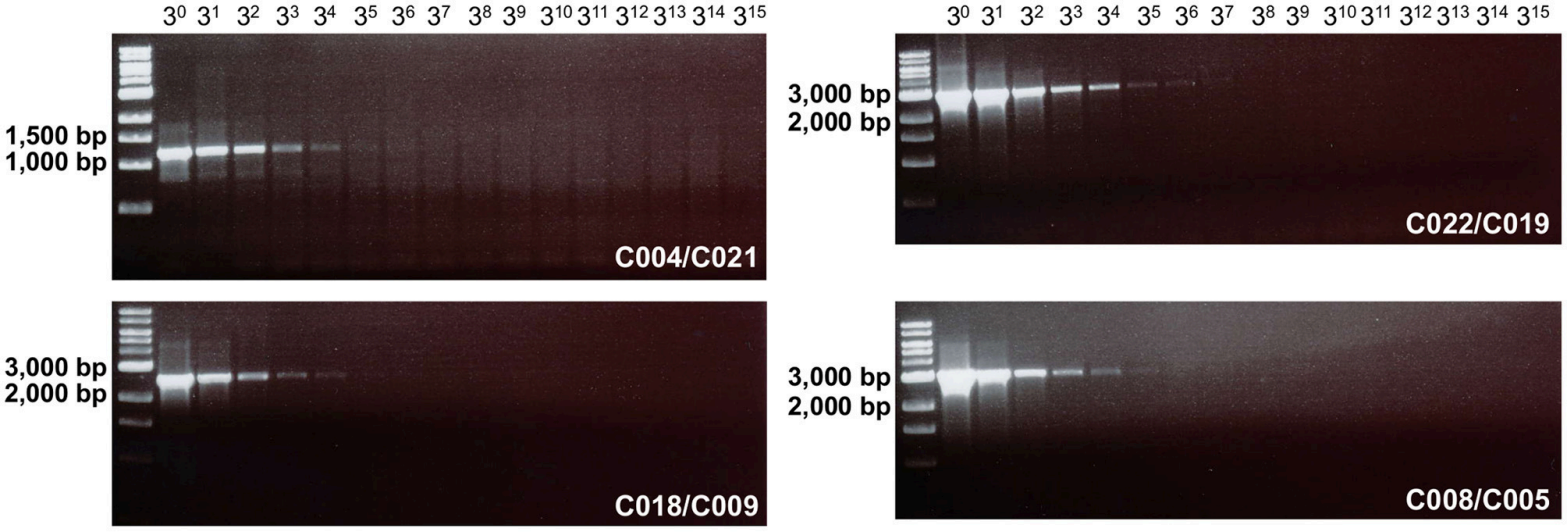
**Sensitivity of the different primer pairs on CV-A13 67001 RNA extracted from three-fold serial dilutions**. The DNA size scale, expressed in base pairs (bp), is indicated on the left side of the gels.

The 2-step RT-PCR assays using C004/C021, C022/C019, C018/C009, and C008/C005 were tested on these extracts. The four primer pairs were able to generate detectable amplicons for RNA extracted from the undiluted supernatant and from the first four dilutions (Figure 3). Since ethidium bromide-stained gels have a detection limit of few nanograms of DNA per band, the absence of visible bands after the amplification of the highest dilutions does not indicate necessarily that no amplicons were generated for these dilutions. Therefore, all the RT-PCR products were investigated through high-throughput sequencing.

After pooling and purification, the RT-PCR products of each dilution were sequenced during the same run. The total number of reads varied from sample to sample, from 88,930 to 316,336 (Table 4). In the sample from the undiluted supernatant, 99.9% of reads mapped against the reference sequence. As expected this proportion decreased when the dilution factor increased but even the samples from the highest dilutions contained a few hundred reads from the virus genome. The non-mapped reads were from cellular nucleic acids. For the six last samples (dilution factor ≥ 3^10^), the number of virus reads was too low to reconstitute the full-length genome sequence through *de novo* assembly. By contrast, *de novo* assembly succeeded in building the full-length genome for all samples with dilution factor ≤ 3^9^, including the last one in which only 2.5% of the reads were from the virus. For this sample, the coverage depth ranged from 5 to 95 along the contig (average of 28). In spite of this relatively low depth, the contig sequence generated for this sample was identical to the Sanger consensus sequence.

These results demonstrated that the method was able to generate full-length sequences of EV-C isolates from a few thousand reads and indicated that sequencing can be attempted even in the absence of detectable bands on agarose gels.

### Detection of mixtures

Supernatants of cell cultures inoculated with clinical and environmental samples often contain mixtures of enteroviruses. In order to determine whether the method was able to sequence mixtures by assembling properly different viral contigs, four mixtures of viruses were prepared by mixing cell culture supernatants of two viruses, 67900 and 67001. These two viruses belong to the same serotype (CV-A13) and display together a nucleotide identity of 83%. The four mixtures contained the same volume of 67900 supernatant but different volumes of 67001 supernatant (Table 5). Thus, the titer ratios ranged from 1:1 to 10:1.

**Table 5:** **Results obtained after sequencing and *de novo* assembly of mixtures of two viruses, CV-A13 67900 and CV-A13 67001**.

**Viral titer (log_10_ TCID_50_)**			**Total number of reads**	***De novo* assembly**			
**67900**	**67001**	**Ratio**		**67900**		**67001**	
				**Reads (%^a^)**	**Average depth**	**Reads (%^b^)**	**Average depth**
7.5	7.5	1:1	267,226	84,674 (31%)	1540	178,860 (66%)	3396
7.5	7.2	2:1	224,848	94,097 (41%)	1689	121,239 (53%)	2313
7.5	6.9	4:1	236,342	129,959 (54%)	2105	104,977 (44%)	1999
7.5	6.5	10:1	147,338	98,121 (66%)	1892	38,977 (26%)	740

a *These percentages indicate the proportion of total reads that were included in the CV-A13 67900 contig through de novo assembly*.

b *These percentages indicate the proportion of total reads that were included in the CV-A13 67001 contig through de novo assembly*.

For the four mixtures, *de novo* assembly generated separate contigs for reads from 67900 and from 67001. As expected, the proportion of reads included in the 67900 contigs increased from mixture 1 to mixture 4 while the proportion of reads in the 67001 contigs decreased (Table 5). Nonetheless, even in mixture 4, which contained 10-fold less starting 67001 RNA compared to mixture 1, the method was able to properly generate a full-length contig corresponding to the expected sequence of 67001.

These results demonstrated that, by using the parameters indicated in Section *De novo* Assembly, the stringency of the *de novo* bioinformatics analysis was high enough to allow the segregation of the reads into separate contigs.

## Discussion

High-throughput sequencing techniques constitute a powerful tool for the study of viruses (Quiñones-Mateu et al., 2014; Nelson and Hughes, 2015). By allowing concomitant sequencing of millions of DNA fragments, they allow rapid sequencing of a great number of samples and in-depth characterization of minority genomic variants. The aim of this study was to develop a convenient method allowing the whole sequencing of the genome of EV-C isolates by using high-throughput sequencing.

Different strategies have been reported to copy viral genomic RNAs into DNA fragments that can be subsequently sequenced by high-throughput techniques.

Some strategies are based on random amplification using non-specific primers (Berthet et al., 2008; Djikeng et al., 2008). These strategies generally lead to the generation of DNA libraries that mainly consist of non-viral sequences, thus decreasing the amount of relevant reads obtained by sequencing. Reducing the amount of unwanted reads requires the use of additional procedures to physically enrich the samples for viral RNAs (Hall et al., 2014) or to limit the amplification of host nucleic acids (Ge et al., 2015). Whole sequencing of virus RNA genomes amplified by random primers can also be impaired by the relatively low amplification rate of some genomic regions, which can lead to gaps in the genomic sequences (Rosseel et al., 2013).

Alternate strategies are based on primers that specifically target the viral RNAs to be sequenced. Such strategies have been already reported to sequence several positive-strand RNA viruses, including enterovirus A71 (Wright et al., 2011; Baronti et al., 2015; Cruz et al., 2016; Thomson et al., 2016). In our experiments, using EV-C-targeting primers rather than random primers limited the number of reads from cellular nucleic acids: the proportion of reads from viral origin was higher than 90% for most samples. The method was thus sensitive enough to determine full-length genomes through *de novo* assembly from only a few thousand reads.

The four primer pairs were efficiently tested on viruses belonging to EV-C types commonly isolated during epidemiological studies, including the three serotypes of PV. They also amplified CV-A1 and CV-A19, which were more closely related to EV-C types recently identified, such as EV-C113 and EV-C116 (Tokarz et al., 2013). Since the primers were designed from an alignment comprising the sequences of all currently known EV-C types, they are likely to be used successfully to amplify any EV-Cs. We cannot exclude that these primers are also able to amplify genomic sequences of viruses closely related to EV-Cs. In particular, the primer C008 targets the *cis*-acting replication element that is highly conserved among EV-As, -Bs, -Cs, and –Ds (Cordey et al., 2008). Reads from EV-As, -Bs, or –Ds, or even rhinoviruses could thus be found in the sequencing data generated from EV-Cs cell cultute supernatants co-infected by members of these species. Nonetheless, as our bioinformatics analyses was stringent enough to discriminate reads from two EV-Cs belonging to the same type, they are likely to discriminate reads from more divergent viruses, thereby preventing the generation of contigs made of reads from viruses belonging to different species.

Our method was able to generate sequencing data even when no bands were observed on agarose gel after the RT-PCR step. This is a great advantage compared to Sanger-based sequencing that requires substantial amounts of DNA. Since the sensitivity of high-throughput sequencing depends as much on the sequencing depth (i.e., the total number of reads obtained for a given sample) as it does on the amount of virus genome copies in the sample, increasing the sequencing depth would overcome the low yield of RT-PCR amplification that could be observed for some samples. Another advantage of high-throughput sequencing compared to Sanger-based technics is that no gel-purification of the RT-PCR products was required, even in the presence of contaminating bands. In case of numerous samples to be analyzed, the RT-PCR products could be purified faster by using ultrafiltration-based or silica-based 96-well plates rather than individual silica-based columns.

For all samples, the coverage depth was heterogeneous along the genome. Heterogeneous coverage depths are often observed after high-throughput sequencing, partly because of the processing of the samples (Head et al., 2014; van Dijk et al., 2014). Thus, biases can be introduced by the random shearing of the DNA being sequenced (Poptsova et al., 2014) and by the PCR amplification performed during the library preparation (Aird et al., 2011). After these steps, some genomic regions can be overrepresented whereas others are underrepresented in the final libraries. Therefore, the coverage depth along the genome does not reflect necessarily the relative abundance of DNA amplicons in the original sample. However, in our experiments, the low coverage depth observed in some genomic regions did not impair the generation of the full-length consensus sequence of the corresponding genomes through *de novo* assembly.

In conclusion, we developed a set of generic primers for the synthesis of RT-PCR products that span the whole genome of EV-C isolates. This method was evaluated by using a panel of viruses already characterized by Sanger-based methods to allow the comparison of the sequences generated by our technique with those obtained previously. For *de novo* assembly, the raw data were analyzed in real conditions, i.e., with no reference to the sequences already obtained by Sanger-based methods. After assembly, the consensus sequences generated in this way were identical to the Sanger consensus sequences.

In this work, the RT-PCR products were sequenced by the Illumina sequencing technology but could be sequenced on any high-throughput sequencing platform. Thus, the sequencing of DNA amplicons covering the whole genome of enterovirus A71 isolates on an Ion Torrent Personal Genomic Machine System was previously reported (Baronti et al., 2015).

This method will serve as a multipurpose tool for laboratories involved in the enterovirus surveillance. It could be used to quickly determine full-length genomic sequences of EV-Cs isolated in cell cultures during environmental surveillance or from patients. In particular, characterization of the genome of vaccine-derived PV, which generally display recombinant genomes made of PV and non-PV genetic sequences (Combelas et al., 2011), could be achieved quickly. The method could also be used to get the full-length sequences of EV-Cs belonging to uncharacterized collections, for example those constituted by the laboratories involved in diagnosis or environmental surveillance (Zaidi et al., 2016). Analyzing large panels of full-length EV-C genomes originating from such collections would help to describe the recombination events that occur between co-circulating viruses and to better understand how recombination drives EV-C evolution.

## Author contributions

Conceived and designed the experiments: MB and FD. Performed the experiments: MB, SS, MJ, PP. Analyzed the data: MB. Contributed reagents/materials/analysis tools: RR, RV, NJ. Wrote the manuscript: MB, FD. Critical revision: MA, BB, NJ.

## Funding

This work was supported by the *Institut Pasteur* (PTR 484), the *Fondation Total*, and the US Department of Health and Human Services (grant No. 5 IDSEP140020-02-00). Patsy Polston is granted by a Pasteur Foundation's Gillings Pasteur Fellowship.

### Conflict of interest statement

The authors declare that the research was conducted in the absence of any commercial or financial relationships that could be construed as a potential conflict of interest.
